# ESF Workshop ‘Proteomics: Focus on Protein Interactions’

**DOI:** 10.1002/cfg.98

**Published:** 2001-10

**Authors:** Gianni Cesareni

**Affiliations:** Department of Biology University of Rome Tor Vergata Via della Ricerca Scientifica Rome 00133 Italy


**Comparative and Functional Genomics**
*Comp Funct Genom* 2001; **2**: 296–297.
**DOI:** 10.1002/cfg.98

**Conference Editorial**

### ESF workshop 'Proteomics: Focus on protein interactions'

Gianni Cesareni*
*Department of Biology, University of Rome Tor Vergata, 00133 Rome, Italy*

**Correspondence to:
G. Cesareni, Department of Biology, University of Rome Tor Vergata, Via della Ricerca Scientifica, 00133 Rome, Italy.
E-mail:
Giovanni.Cesareni@uniroma2.it*

Approximately 70 scientists met, from May 11–13 2001, in Villa Mondragone in the hills south of Rome to discuss strategies to describe the complete protein interaction network inside a cell. Very few of the participants still needed to be convinced that this is an essential step if we want to try to interpret the functional information contained in genomic databases. This was well accepted before the workshop started. The discussion revolved around how this can be achieved most effectively and which methods we should focus on if we want to get reliable biological information. Most of the high throughput methods that are currently used in large genomic protein interaction projects were represented by at least one of the 17 invited speakers. For the sake of simplicity the workshop presentations were divided into four sessions encompassing genetic methods, protein and peptide arrays, mass spectrometry and bioinformatic methods.

Pierre Legrain, whose presentation is reported in more detail on page 301, focussed on the critical comparison of the different approaches that have been utilized in recent large scale 2-hybrid interaction screenings. Particularly surprising, and perhaps disappointing, is the finding that two large projects that aimed at deciphering the complete protein interaction map in *S. cerevisiae* show only a 15% overlap and recapitulate no more than 13% of the published interactions detected by the community of yeast biologists. Pierre Legrain suggested that an approach based on the expression of protein fragments, instead of full-length proteins, might contribute to decreasing the number of false negatives, as demonstrated in the *Helicobacter pylori* protein interaction project. Andreas Pluckthun and Brian Kay described two alternative methods, ribosome display and phage-display, that have a genomic potential. Ribosome display, although still in a development phase, holds great promises since it offers the potential to screen a number of partners that is by three to four logs larger than conventional display methods. Panning of peptide repertoires of random sequence displayed on filamentous phage capsids, on the other hand, not only permits one to infer the identity of natural protein partners but also allows precise mapping of the interaction sites (reviewed on page 304). Furthermore, this approach provides leads to develop molecules that, by binding at high affinity to either partner, disrupt the formation of a protein complex in a cell. Genetic methods are selective, although a large-scale screen in an array format has been described. By this approach each single interaction is tested independently and problems due to selective growth disadvantage of specific clones may be overcome.

The array approach can be better implemented when proteins or peptides are orderly spotted or synthesized on solid supports, for instance a cellulose membrane, or a glass slide, as in DNA array. The technology of protein chips is far from being as accessible to the non-specialised laboratory as DNA chip technology. The problems to be overcome range from the difficulties experienced in

Copyright © 2001 John Wiley & Sons, Ltd.

assembling large collections of soluble proteins or antibodies, to the background that is often observed in the screening procedure. The promises and the pitfalls of this approach are discussed in some detail in the accompanying discussion paper by Michael Taussig (see page 298). A similar approach, based on the synthesis of large numbers of relatively short peptides on a cellulose membrane, was presented by Jens Schneider-Mergener (reviewed on page 307). The technology originally developed by R. Frank has now reached maturation and has turned into a powerful approach to look at protein interactions. Experiments were presented which demonstrated that, by combining the identification of a rough consensus ligand motif by phage display with scanning for those peptides in a whole proteome that contain the consensus motif, it is possible to identify physiological partners of any given protein binding module. The approaches described so far are sometimes collectively referred to as 'bottom up' approaches since they all rely on the synthesis, *in vitro* or *in vivo*, of a collection of translation products to be tested for potential interactions. The set of derived interaction networks represents the inferred protein network in a sort of virtual cell.

Far more direct are the 'top down' approaches that start from real cells and aim to purify, mainly by affinity capture, and characterise native complexes. This approach has received a boost in the past few years because of the development of very sensitive mass spectrometric methods that permit precise determination of the mass of tiny amounts of proteins separated by gel electrophoresis. Furthermore, as exemplified in the manuscript by Mattei *et al*., (next issue) this approach can also deal with the characterisation of protein interactions that are mediated by complex protein modifications. Large-scale proteomic projects are now possible. Giulio Superti-Furga reported on a project that aims at characterizing all the protein complexes in a yeast cell. The project should be finished by the end of the year. It would be interesting to find out which percentage of the interactions described by the community of yeast scientists will be rediscovered by this large-scale approach and how many new interactions will be added to the yeast 'interactome'.

Finally, the topics discussed in the bioinformatics session covered database integration, prediction of functional protein partners (see the review by Manuela Helmer-Citterich, page 314) and data mining by analysis of the textual information contained in the databases of scientific abstracts (reviewed on page 310).

Most of us are interested in revealing the set of functional interactions occurring inside the cell. At this stage, the 'top down' approach, by revealing the protein composition of native complexes that are present in the cell, is likely to provide a wealth of biologically reliable information. It is not clear, however, which percentage of physiologically relevant interactions occur at an affinity, molarity or concentration that are beyond the limits of detection by silver staining and/or mass spectrometry. 'Bottom up' approaches are not limited by these factors. Another goal in the field is the mapping of the binding surfaces in the interaction partners and the development of molecules that, by binding with high affinity to either partner, disrupt the formation of the complex *in vivo* and permit the analysis of the physiological consequences of this disruption. Genetic methods and screening of large collections of peptide and protein arrays will still have a say in this area. The take-home lesson is that it is safe, for the genomic programs, not to restrict the investment to a limited number of approaches. Each approach discussed at the workshop at the European Science Foundation in Villa Mondragone has the potential to give a sizeable and largely complementary contribution to the goal of assembling a protein interaction network.

Copyright © 2001 John Wiley & Sons, Ltd.

